# *In Vitro* Immune Toxicity of Depleted Uranium: Effects on Murine Macrophages, CD4^+^ T Cells, and Gene Expression Profiles

**DOI:** 10.1289/ehp.8085

**Published:** 2005-08-17

**Authors:** Bin Wan, James T. Fleming, Terry W. Schultz, Gary S. Sayler

**Affiliations:** 1 Center for Environmental Biotechnology,; 2 Department of Ecology and Evolutionary Biology,; 3 Department of Microbiology, and; 4 Department of Comparative Medicine, University of Tennessee, Knoxville, Tennessee, USA

**Keywords:** apoptosis, CD4^+^ T cell, cytokine gene expression, depleted uranium, macrophage function, necrosis

## Abstract

Depleted uranium (DU) is a by-product of the uranium enrichment process and shares chemical properties with natural and enriched uranium. To investigate the toxic effects of environmental DU exposure on the immune system, we examined the influences of DU (in the form of uranyl nitrate) on viability and immune function as well as cytokine gene expression in murine peritoneal macrophages and splenic CD4^+^ T cells. Macrophages and CD4^+^ T cells were exposed to various concentrations of DU, and cell death via apoptosis and necrosis was analyzed using annexin-V/propidium iodide assay. DU cytotoxicity in both cell types was concentration dependent, with macrophage apoptosis and necrosis occurring within 24 hr at 100 μM DU exposure, whereas CD4^+^ T cells underwent cell death at 500 μM DU exposure. Noncytotoxic concentrations for macrophages and CD4^+^ T cells were determined as 50 and 100 μM, respectively. Lymphoproliferation analysis indicated that macrophage accessory cell function was altered with 200 μM DU after exposure times as short as 2 hr. Microarray and real-time reverse-transcriptase polymerase chain reaction analyses revealed that DU alters gene expression patterns in both cell types. The most differentially expressed genes were related to signal transduction, such as *c-jun*, *NF-*κ *Bp65*, neurotrophic factors (e.g., *Mdk*), chemokine and chemokine receptors (e.g., *TECK*/*CCL25*), and interleukins such as *IL-10* and *IL-5*, indicating a possible involvement of DU in cancer development, autoimmune diseases, and T helper 2 polarization of T cells. The results are a first step in identifying molecular targets for the toxicity of DU and the elucidation of the molecular mechanisms for the immune modulation ability of DU.

Depleted uranium (DU) is a by-product of the enrichment process of natural uranium (Priest 2001). The release of uranium into the environment presents a threat to human and ecologic health in many parts of world (Hass et al. 1998; Murray et al. 2002). DU shares chemical properties with natural or enriched uranium, but the major hazard rendered by DU results from its heavy metal toxicity rather than from radiologic toxicity (Fisenne and Welford 1986; Priest 2001). The adverse health effects of DU compounds are partially dependent on its chemical form. Uranium compounds in +2 to +4 valence states are essentially insoluble. However, *in vivo* soluble uranium is always hexavalent, regardless of the oxidation state of uranium compound taken up (Edison 1994). It is this form (+6) that is of toxicologic importance. Because of their high affinity for phosphate, carboxyl, and hydroxyl groups, uranyl compounds readily combine with proteins and nucleotides to form stable complexes (Moss 1985).

Serum uranium forms a variety of nondiffusible complexes such as uranium–albumin compounds and diffusible ones such as ionic uranyl hydrogen carbonate complex (Moss 1985). Although the most characteristic response to DU exposure either short or long term is renal dysfunction (Domingo 1995; Leggett 1989; Zamora et al. 1998), uranium is also localized within the central nervous system, testes, lymph nodes, and spleen, suggesting the potential for uranium to cause health problems at these sites (Domingo 2001; Pellmar et al. 1999; Wrenn et al. 1985). Uranium-induced pathological changes in the testes and thyroid glands have been documented (Malenchenko et al. 1978).

*In vitro* studies have examined the effects of DU on a variety of cell types. For example, Chinese hamster ovary cells exposed to DU exhibit lower cell viability, depressed cell cycle kinetics, and increased sister chromatid exchanges, micronuclei, and chromosomal aberrations after DU exposure (Lin et al. 1993). Kidney cells release lactate dehydrogenase upon uranium exposure (Furuya et al. 1997), whereas human osteoblast cells are transformed to a neoplastic phenotype after *in vitro* DU exposure (Miller et al. 1998).

More important to this investigation, some studies indicated that immune cells are also involved in DU toxicity. Macrophages can actively internalize the uranium, with the subsequent occurrence of cell apoptosis (Kalinich and McClain 2001; Kalinich et al. 2002). Other evidence suggests the involvement of cytokine gene expression in DU toxicity, and the changes of some of these genes are associated with immune responses. For example, recent studies demonstrated that DU induces abnormal expression and release of tumor necrosis factor (TNF) and interleukin (IL)-6 in macrophages (Gazin et al. 2004: Zhou et al. 1998).

During the Gulf War, tons of DU weapons were fired, and DU shrapnel was permanently embedded in the bodies of many soldiers (sometimes removing shrapnel is fatal). In addition inhalation of DU combustion particles on the battlefield is also a major source of exposure to high concentrations of DU. It was hypothesized that Gulf War syndrome may be explained as a systemic shift in cytokine balance from a T helper (Th) 1 profile toward a Th2 profile because the syndrome is clinically similar to autoimmune diseases (Rook and Zumla 1997; Skowera et al. 2004). In this study we hypothesized that DU exposure may compromise the immune system function by inducing immune cell apoptosis and modulating immune cell cytokine gene expression, which may be predictive of DU immunotoxicity. This hypothesis is consistent with the findings of Li et al. (2001), Pallardy et al. (1999), and Rodenburg et al. (2000), which showed that cell death through apoptosis or necrosis may cause serious adverse effects such as immunosuppression or lead to an altered immune response. More specifically, because of the macrophage’s phagocytosis activity and ubiquitous presence throughout the body, it is also important to assess the effect DU may have on macrophage function as accessory cells to T-lymphocyte activation/proliferation. Cytokine gene expression profiling of DU-exposed immune cells should contribute to the understanding of the molecular mechanisms of DU toxic effects on the immune system. To test the above hypotheses, we exposed macrophages and primary CD4^+^ T cells to DU (in the form of uranyl nitrate) and examined for evidence of apoptosis and altered macrophage function in promoting lymphocyte proliferation. Macrophages and T cells were also exposed to DU at noncytotoxic concentrations, and the effect of DU-modulated cytokine gene expression was examined. The results of these experiments suggest a possible role for DU in carcinogenesis and autoimmune diseases.

## Materials and Methods

### Chemicals.

Uranyl nitrate [UO_2_(NO_3_)_2_ · 6H_2_O], with a specific activity of approximately 0.2 μCi/mg, and sodium nitrate (NaNO_3_) were purchased from Mallinckrodt Specialty Chemicals Co. (Phillipsburg, NJ) and both were dissolved in water. Lipopolysacchride and concanavalin A (ConA) were from Sigma (St. Louis, MO) and were dissolved in DMSO. [α-^33^P]-Deoxyadenosine 5′-triphosphate was purchased from ICN Radiochemicals (Costa Mesa, CA).

### Animals.

BALB/c and DO11.10 T-cell receptor (TCR)–transgenic mice were originally obtained from The Jackson Laboratory (Bar Harbor, ME) and bred and housed under pathogen-free conditions in the animal care facility at the University of Tennessee, Knoxville, according to the animal protocol procedures approved by the Committee on the Care of Laboratory Animal Resources. Mice 6–8 weeks of age were used for cell preparation.

### Cell preparations.

Collected peritoneal elicited macrophages were collected and pooled from three to four Balb/c mice injected intraperitoneally with thioglycollate (TG) broth (3% wt/vol; 1 mL/mouse; Difco Laboratories, Livonia, MI) 4 days before cell collection. Cells were plated onto 25 cm^2^ Corning cell culture flasks or polystyrene six-well flat-bottom microtiter plates and were incubated at 37°C, 5% CO_2_/95% air, and 95% humidity for 4 hr to allow the macrophages to adhere to the surfaces. The surfaces were washed twice with warm PBS to remove all nonadherent cells, and the macrophage layer was cultured overnight in complete RPMI-1640 (cRPMI-1640) containing 10% low-endotoxin, heat-deactivated fetal bovine serum (Sigma, Copenhagen, Denmark), 10^–5^ M 2-mercaptoethanol, L-glutamine (20 mM), and penicillin and streptomycin (100 U/mL each). The resulting macrophage purity was > 95%, determined by CD11b staining analysis. Cells were then washed twice, stained with trypan blue exclusive dye, and counted. The cells were exposed to uranyl nitrate at various concentrations in 25-cm^2^ flasks (for RNA isolation) or six-well plates (for flow cytometry analysis).

Splenic CD4^+^ T cells were negatively isolated using the magnetic activated cell sorting method according to the manufacturer’s protocols (Miltenyi Biotec, Auburn, CA). In brief, pooled splenic cells from three to four DO11.10 mice were stained with a cocktail of biotin-conjugated monoclonal antibodies against CD8a(Ly-2) (rat IgG2a), CD11b (Mac-1) (rat IgG2b), CD45R (B220) (rat IgG2a), DX5 (rat IgM), and Ter-119 (rat IgG2b). The mixture was then incubated with Anti-Biotin MicroBeads (Miltenyi Biotec) and the cell suspension was passed through an LS magnetic separation column (Miltenyi Biotec). The major cell composition of elute is CD4^+^ T cells (> 95%). After washing, the cell density was adjusted to 1 × 10^6^ cells/mL cRPMI-1640 media and the DU exposure was performed in anti-CD3–coated 96-well plates, followed by RNA isolation or flow cytometry analysis. Fresh cells from new living animals were purified each time the assays were repeated; three individual experiments were performed.

### Cell staining and flow cytometry analysis of cell death.

For both macrophages and primary CD4^+^ T cells, cell death analysis using flow cytometry was performed in triplicate. We handled and treated cells according to the protocol provided with the Annexin-V–fluorescein (A-V–FITC) Apoptosis Detection Kit (Sigma, Copenhagen, Denmark). Cells (1 × 10^6^ cells/mL) were exposed to uranyl nitrate at 0–200 μM (macrophages) or 0–500 μM (CD4^+^ T cells). The cells were harvested and washed once with PBS and resuspended in 1 × binding buffer. A 500-μL aliquot of the cell suspension was stained with 5 μL of A-V–FITC and 10 μL of propidium iodide (PI) in a 12 × 75 mm test tube for 10 min at room temperature, protected from light. We then analyzed cells using the flow cytometry FACScan (BD Biosciences, San Jose, CA) by counting 30,000 events. The data files were saved automatically by CellQuest software (BD Biosciences), and WinMDI (version 2.8; The Scripps Institute, Flow Cytometry Core Facility, La Jolla, CA), was used to perform quadrant analysis. A two-tailed *t*-test was performed to determine the significant difference between treatment and control experiments using Excel (Microsoft, Redmond, WA).

### Lymphoproliferation assay.

We used a T-lymphocyte proliferation assay to estimate the macrophage function as accessory cells. The exposure follows the model described by Krocova et al. (2000): elicited peritoneal macrophages were placed into the wells of 96-well microtiter plates (Corning Inc., Corning, NY), allowed to adhere for 4 hr, and then exposed to cRPMI-1640 media containing DU (10, 50, 100, 200, 500, and 1,000 μM) or an equal amount of medium with no DU added. After 2 hr incubation, the cells were washed, and then the purified CD4^+^ T cell suspension (1 × 10^6^ cells/mL with 5 μg/mL ConA) was added to each well containing treated adherent macrophages. Simultaneously, we set up non–T-cell controls by replacing the T cells with same amount of culture medium. The plate was incubated at 37°C, 5% CO_2_/95% air, and 95% humidity for 48 hr. Then, we added 10 μL of MTT [3-(4,5-dimethylthiazolyl-2)-2,5-diphenyltetrazolium bromide] solution (5 mg/mL) to each well, and allowed the plate to incubate for an additional 4 hr. At the end of the incubation, we added 100 μL of acidic isopropanol (0.04 M HCl in absolute isopropanol) and mixed to dissolve the converted dye formazan. The absorbance data were recorded by a spectrophotometer (Bio-Tek Instruments, Inc., Winooski, VT) at 562 nm.

### Mouse cytokine cDNA microarray analysis.

The Panorama mouse cytokine gene array (Sigma, St. Louis, MO) consisting of 514 different cytokine-related cDNAs printed onto charged nylon membranes was used to analyze gene expression profile. A detailed description and a list of genes included on the array can be found on the Sigma-Genosys website (Sigma 2004). Briefly, we exposed macrophages and CD4^+^ T cells to 50 and 100 μM DU, respectively, for 24 hr; after treatment, we extracted total RNA from each sample using Trizol reagent and treated the RNA with RNase-free DNase I (Gibco-BRL Life Technologies Inc., Grand Island, NY). Using mouse cytokine gene cDNA-labeling primers (Sigma-Genosys, St. Louis, MO), 2 μg RNA were reverse transcribed to generate a [α-^33^P]-dATP–labeled cDNA probe. We removed unincorporated nucleotides from the probe using NucTrap probe purification columns (Stratagene, La Jolla, CA). EDTA was added to bring the final concentration to 10 mM, and the probe was heat denatured at 95°C for 5 min. Arrays were hybridized with probes in ULTRArray hybridization buffer (Ambion, Inc., Austin, TX) overnight at 55°C. The arrays were then washed extensively at 50°C under both low-and high-stringency conditions [2 × saline sodium citrate (SSC), 0.5% SDS for low-stringency wash solution, 0.5 × SSC, 0.5% SDS for high-stringency wash solution] for 2 × 30 min. The membranes were air dried, sealed in a clear plastic bag, and exposed to low-energy storage phosphoimage screens (Kodak, Rochester, NY). The images were scanned at 50-μm resolution on a Storm 840 PhosphorImager (Molecular Dynamics, Inc., Sunnyvale, CA). The image files were analyzed using ArrayVision software (version 6.0; Imaging Research, St. Catharines, Ontario, Canada), and the numerical output was exported in Microsoft Excel format to ArrayStat (version 1.0; Imaging Research Inc.) for statistical analysis. Microarray data obtained here are available at Gene Expression Omnibus (GEO 2005; accession no. GSE2333).

### Real-time reverse-transcriptase PCR analysis.

Real-time reverse-transcriptase polymerase chain reaction (RT-PCR) was used to verify gene expression of microarray analysis. The assay was performed in triplicate in a DNA Engine Opticon system (MJ Research Inc., Waltham, MA) using SYBR Green I as the detection format (Qiagen, Inc, Valencia, CA). First, we converted total RNA to first-strand cDNA using reverse transcription, and then performed real-time RT-PCR analysis using the SYBR Green PCR kit (Qiagen). The PCR primers are listed in Table 1. After PCR, we performed melting curve analysis and visualized the PCR products using gel electrophoresis to assess the specificity of PCR amplification reactions. For both the reference gene [glyceralde-hyde-3-phosphate dehydrogenase (*GAPDH*)] and test gene, we constructed standard curves and determined the slope to calculate the PCR efficiency according to Pfaffl (2001; Pfaffl et al. 2002). We calculated differences in gene expression between treatment and control using PCR efficiencies and threshold cycle numbers (Ct values), which were normalized against *GAPDH*. The formulation used for calculating the ratio of gene expression between control and treatment groups is described by Pfaffl (2001; Pfaffl et al. 2002) as

[1]
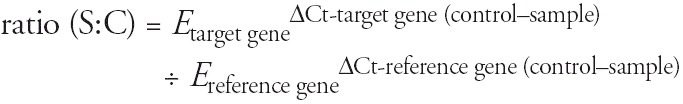


where ratio(S:C) is the expression ratio of DU-treated sample over control; *E*_target gene_ is the PCR efficiency of target gene; *E*_reference gene_ is the PCR efficiency of reference gene; ΔCt-target gene (control–sample) is the difference of target gene Ct values between control (*C*) and DU-treated samples (*T*); and ΔCt-reference gene (control–sample) is the difference of reference gene Ct values between control and DU-treated samples.

### Statistics.

Data were expressed as the mean ± SD, and a Student *t*-test was used to compare the differences between treatment and control groups, with the significance level set at *p* < 0.05. For microarray data analysis, blots were normalized to the mean values of the entire array with background subtraction. Three sets of data were generated from three biologic replicate experiments; if there were only one valid observation for a gene within a condition, that datum was disregarded for analysis. The outliers were detected by examining standardized residuals automatically using ArrayStat software. A curve-fit random error estimate method was employed for a proportional model with offset. The data were transformed logarithmically, and a *Z*-test for two independent conditions was performed according to Benjamini and Hochberg (1995). Differentially expressed genes were identified on the basis of the significance level (*p* < 0.05 or effective *p* < 0.05/number of analyzed genes).

## Results

### Macrophage cell death.

We determined induction of apoptosis in macrophages using flow cytometry analysis of cells labeled with A-V–FITC. A-V protein can bind, by a calcium-dependent process, to phosphatidylserine (PS) presented on the surface of cells undergoing apoptosis (Bertho et al. 2000). PS is normally sequestered on the inner leaflet of the plasma membrane. However, during apoptosis, membrane phospholipid asymmetry is lost and PS is exposed to the outer leaflet, where it can interact with A-V. Cells that stain positively for A-V and negatively for PI are considered cells in an early stage of apoptosis, whereas those that stain positively for both indicators either are in late apoptotic stage or are undergoing necrotic cell death.

**Figure 1:**
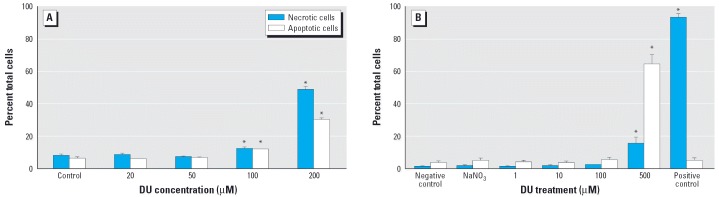
Cell apoptosis and necrosis under DU exposure. (*A*) Macrophages were treated with 0 (control), 20, 50, 100, or 200 μM DU for 24 hr. (*B*) CD4^+^ T cells were treated with 0 (negative control), 1, 10, 100, or 500 μM DU, 1 mM NaNO_3_, or 1 μg/mL staurosporine (positive control) for 24 hr. Data are presented as percentage of cells in the apoptotic or necrotic state and are the means of triplicate experiments. Error bars represent SD. *Difference from negative control is statistically significant (*p* < 0.05).

The treatment of macrophages with DU as uranyl nitrate resulted in apoptotic cell death. As shown in Figure 1A, treatment with 20 and 50 μM DU for 24 hr did not cause an apparent increase in A-V and PI staining. However, 100 and 200 μM DU treatment led to a significant increase of both A-V and PI staining (*p* < 0.05), with the percentages of A-V binding at 12.3 and 30.5%, respectively, and the percentage of PI positive cells at 12.4 and 49.2%, respectively. The results indicated that both cell apoptosis and necrosis increase with an increase in concentration. DU at 50 μM was determined as noncytotoxic to macrophages.

**Figure 2:**
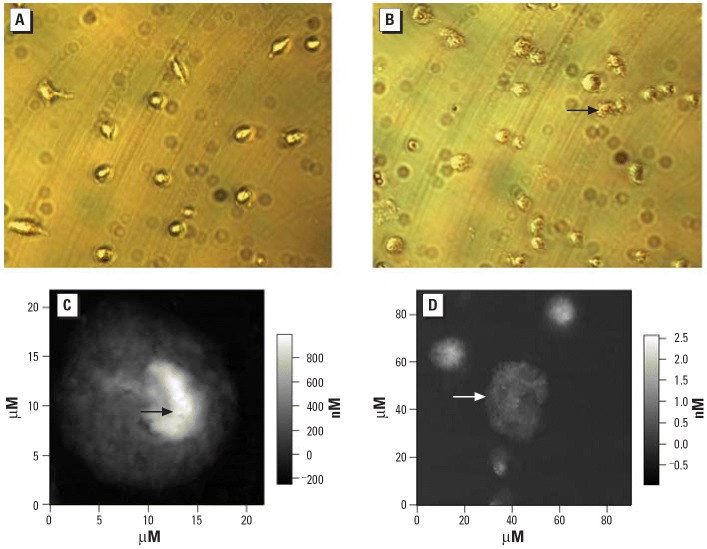
Representative bright-field and atomic force photomicrographs of macrophages with or without DU exposure. The cells were cultured in medium without or with 100 μM DU (as uranyl nitrate) for 24 hr and then processed for microscopy. Bright-field images (40 × magnification) of control (*A*) and DU-treated cells (*B*); the rough membrane structure of DU-treated cells was shown in *B*. Atomic force microscopy images of single control (*C*) and DU-treated cells (*D*), with or without nucleus area, respectively. The apoptotic body is presented in *D* as smaller separated bodies. The arrows in *B* and *D* indicate cells undergoing apoptosis after 24 hr of 100 μM DU exposure. Arrow in *C* indicates the nucleus feature of a normal cell.

Using light microscopy and atomic force microscopy, we investigated the morphologic changes in macrophages treated with 100 μM DU for 24 hr (Figure 2). We treated the macrophages with 0 or 100 μM DU for 24 hr and fixed the cells by soaking them in 2% formaldehyde (prepared in propanol), followed by incubation in 0.1% Triton-X 100 for 15 min. We then air dried the cells at room temperature in preparation for imaging by atomic force microscopy. Alternatively, cells after DU exposure were directly imaged by light microscopy. When adherent macrophages undergo necrosis or cellular disintegration, adherent cells will disassociate and float in the medium. Figure 2 shows that the apoptotic cells are still adherent to the surface and show the rough shape of the cell membrane and no apparent nuclear structure (Figure 2D). In the atomic force microscopy images, the darkness represents the height of sample surface over the vessel surface. Note that in Figure 2C, which is a normal cell, the thicker area (brightest) is the nucleus of the cell, but in Figure 2D, there is no such area, which is indicative of loss of nuclear structure. In Figure 2D, the small particle-like areas indicate apoptotic bodies.

### CD4^+^ T-cell death analysis.

The treatment of CD4^+^ T cells with DU (as uranyl nitrate) resulted in apoptotic as well as necrotic cell death. As shown in Figure 1B, treatment with 1, 10, and 100 μM DU for 24 hr did not result in significant increase of either A-V staining or PI staining, indicating that these treatments did not induce cell apoptosis or necrosis. However, there was a significant increase of apoptotic and necrotic cells after treatment with 500 μM DU (*p* < 0.05), 64.5 and 15.3%, respectively, whereas the apoptotic and necrotic cell percentages of negative controls were 3.5 and 1.5%, respectively. As expected, the positive controls had a much higher percentage of necrotic cells, whereas the percentages of necrotic and apoptotic cells in the 1 mM NaNO_3_ control group were not different from those of negative control, indicating that apoptosis and necrosis in DU treatment were not attributable to the NO_3_^–^ ion but were due to the uranyl ion. DU at 100 μM was determined as noncytotoxic to CD4^+^ T cells during 24-hr exposure.

### Lymphoproliferation assay.

**Figure 3:**
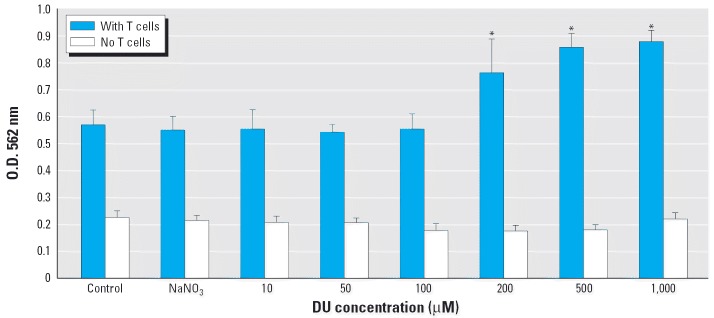
Effect of DU on the accessory cell function of peritoneal adherent macrophages. The effect of DU on lymphocyte (CD4^+^ T cell) proliferation was determined by MTT assay, which indirectly reflects the accessory cell function of macrophages in promoting lymphocyte proliferation. Results are expressed in optical density values read at 562 nm wavelength as mean and SD of triplicate analyses. *Difference from control is statistically significant (*p* < 0.05).

Concanavalin A (ConA) was used to activate T cells. The likely mechanism is that ConA indirectly cross-links the TCR and sends the activation signals. Activation of T cells is also dependent on the presence of non-T cells that function as accessory cells, which provide additional and essential costimulatory signals for T-cell proliferation (Coligan 1991). Exposure to DU at concentrations > 200 μM significantly enhanced the functionality of macrophages, as shown by increased T-cell proliferation under the induction of ConA (Figure 3, solid bars). Lower concentrations of DU < 100 μM did not change the T-cell proliferation. However, 200, 500, and 1,000 μM DU treatment of macrophages significantly (*p* < 0.05) enhanced T-cell proliferation in a concentration-dependent manner, with the optical density measurements in MTT assay increasing from 0.56 (control) to 0.76, 0.86, and 0.87, respectively. In addition, 1 mM NaNO_3_ treatment did not influence the measurement, excluding the contribution of nitrate ions (NO_3_^–^) to the toxic effect on macrophages. In this study, pretreatment of macrophages with various concentrations of DU for 2 hr did not cause significant loss of cells in 96-well plates, as shown by open bars in Figure 3. Therefore, it is safe to preclude the possibility that the alteration of T-cell proliferation is caused by variations in macrophage cell numbers.

### DU influence on gene expression.

Gene expression in macrophages and CD4^+^ T cells under noncytotoxic DU exposure was analyzed by cDNA microarray. The results were from three biologic replicates. Table 2 lists 29 (6% of all analyzed genes) genes whose expressions were significantly (*p* < 0.05) changed in macrophages upon 50 μM DU exposure. Of these 29 genes, 24 (5%) were up-regulated, and 5 (1%) were down-regulated. Although a variety of gene groups are affected, the groups with multiple affected genes include signal transduction–related, cytokine- and IL-related, and apoptosis-related groups. Other genes with altered expression such as *LTBP-2* and *Mdk* are neurotrophic factors or involved in binding protein, respectively.

The differentially expressed genes in CD4^+^ T cells under noncytotoxic (100 μM) DU exposure are listed in Table 3. Although many of the same gene groups are represented, the specific genes, except for *Mdk* listed in Tables 2 and 3 are different. Specifically, chemokine-related genes are up-regulated in CD4^+^ T cells but not in macrophages. Moreover, *IL-5* is related to T-cell functionality.

### Real-time RT-PCR analysis.

We used real-time RT-PCR analysis as a confirmative method for the genes determined to be differentially expressed by microarray analysis and performed the analysis for selected genes in both cell types. Table 4 shows some of the RT-PCR results, along with the corresponding microarray results. RT-PCR analysis showed that under DU exposure, expression of genes such as *Mdk*, *c-jun*, and *IL-10* was enhanced in macrophages, and *Mdk* and *IL-5* in CD4^+^ T cells. The ratios of all genes except for *IL-10* were in accordance with those determined by microarray analysis. We performed the assay in triplicate. Generally, this quantitative RT-PCR assay confirmed the microarray results.

## Discussion

Uranium environmental contamination from mining, processing, and military industries has heightened concern of the possible environmental and health effects of DU exposure. DU can enter the body by ingestion, inhalation, contamination of wounds, and embedded shrapnel (McClain et al. 2001). At the cellular level, accumulation of DU has been observed in various macrophage cell lines (Gazin et al. 2004; Kalinich et al. 2002), and one of the first issues to address is whether DU induces macrophage death and at what level this toxic effect occurs. In this study we used flow cytometry analysis of A-V/PI binding to study apoptosis and necrosis. The results showed that apoptosis and necrosis occurred after 24 hr with DU (as uranyl nitrate) treatments ≥ 100 μM. These results are similar to those observed in a previous study that used uranyl chloride and a macrophage cell line, J774 (Kalinich et al. 2002) and were also comparable with other reported experiments using human osteoblast cells (Miller et al. 1998). Apoptosis and, to a lesser extent, necrosis occurred simultaneously after 24 hr when T cells were exposed to concentrations as high as 500 μM DU. Below 500 μM, apoptosis and necrosis were not observed. CD4^+^ T cells are more resistant than macrophages, which may be because macrophages can actively engulf DU particles (Kalinich et al. 2002), but CD4^+^ T cells do not. Compared with other heavy metals, DU is much less toxic to CD4^+^ T cells than mercury, whereas lead and vanadium have approximately the same toxicity as DU (Shen et al. 2001).

Once the toxicity of DU to immune cells was determined, the issue arose as to how DU affects the function of immune cells. In the presence of ConA, *in vitro* T-cell activation requires accessory cells for co-stimulatory signals (Pollard and Landberg 2001). In the present study we assessed the ability of DU-exposed macrophages to function as accessory cells by measuring the CD4^+^ T-cell proliferation. The results indicated that higher concentrations (200 to ~ 1,000 μM) of DU were able to alter macrophage functionality *in vitro* in a concentration-dependent manner, which led to significant T-cell proliferation. This response is similar but occurs at higher concentrations compared with other heavy metals such as lead, which induces lymphocyte proliferation at a concentration range of approximately 12–120 μM (Krocova et al. 2000). The results in this study demonstrated that a short-term, high-concentration DU exposure was able to perturb rapidly the interaction between macrophages and T cells, and immune function.

Previous studies on the toxic effects of heavy metals, including uranium, indicated the involvement of cytokine regulation in immunomodulatory activities (Gazin et al. 2002; Krocova et al. 2000). However, these studies focused on the expression of only a few cytokine genes such as interleukins, *NF-*κ*B*, or *TNF-*α. Global gene expression analysis in kidney tissue after DU exposure suggested that genes involved in multiple biologic functions, including signal transduction, may be altered by uranium exposure (Taulan et al. 2004). We further asked what effects DU might have on the immune system if the exposure scenario were nonlethal and long term and how it might relate to cytokine gene expression.

In this present study we used a mouse cytokine gene array, and as expected, genes related to signal transduction pathways were significantly modulated by DU exposure (Tables 2, 3). In DU-exposed macrophages the most highly expressed gene was *NF-*κ *B p65* (Table 2). Miller et al. (2004) demonstrated that DU (5–50 μg/mL or 18.5–185 μM) had profound influences on multiple signaling pathways in HepG2 cells; interestingly, the genes they identified also related to the NF-κ B pathway, as indicated in our research. The important role of NF-κ B in uranium toxic effects has been reported previously by Gazin et al. (2002). Our results provide direct evidence showing that DU is able to activate NF-κ B by increasing the expression of the p65 subunit. The NF-κ B family of transcription factors not only are key regulators of genes involved in immune and inflammatory reactions (Li et al. 2001; Tak and Firestein 2001) but also are involved in many aspects of cell growth, differentiation, and proliferation via the induction of certain growth and transcription factors (e.g., c-myc, ras, p53). The co-induction of *NF-*κ *B*, *MMP-13*, and *c-myc* indicated in our microarray results is consistent with previous work by Tak and Firestein (2001).

NF-κ B can mediate both inflammatory and antiinflammatory responses by regulating genes encoding either proinflammatory or antiinflammatory activities (e.g., *IL-10*) (Baldwin 2001; Bierhaus and Nawroth 2003; Xu and Shu 2002). In our microarray analysis, the latter was indicated by the up-regulation of *IL-10* gene in macrophages upon DU exposure (Table 2). Activation of NF-κ B requires degradation of I-κ B (nucear factor of kappa light chain gene enhancer in B-cells inhibitor, β ) with the help of I-κ B kinases, the activity of which depends on binding with NF-κ B–inducing kinase (NIK) (Chen et al. 2001; Wooten 1999). The activation of NIK as shown in this study (Table 2) supports the conclusion that the NF-κ B signaling pathway was adopted by macrophages under the DU exposure. DU may induce NIK activity leading to the up-regulation of NF-κ B (by increasing the p65 subunit level), which further activates expression of a variety of cytokine genes, such as *c-myc* and *MMP-13*, as indicated in our array data. This hypothesis is supported by the study of Miller et al. (1998) that demonstrated DU-induced tumorigenic activity in osteoblast cells.

It is interesting to note that the expression of the neurotrophic factor Mdk was highly induced in both primary macrophages and CD4^+^ T cells (Tables 2, 3) after DU exposure. *Mdk* gene expression is restricted to only a few types of cells such as kidney and epithelial cells (Garver et al. 1993; Hu et al. 2002); it is very unusual that this gene was regulated by DU in immune cells. To our knowledge [main references were Tully et al. (2000) and Yamada and Koizumi (2002)], the up-regulation of *Mdk* by heavy metal exposure has not been previously reported, which may indicate a common mechanism of DU immunotoxicity to both macrophages and T cells and may provide a biologic marker for DU exposure. Because *Mdk* levels often increase in the early stage of cancer progression, it has been suggested as a tumor marker (Muramatsu 2002). High induction of *Mdk* expression in this study presents further evidence for the possible involvement of DU in carcinogenesis, as reported by Miller et al. (1998).

A DU-induced Th1–Th2 shift has been long postulated to play a role in the development of Gulf War syndrome (Rook and Zumla 1997; Skowera et al. 2004). The complex balance between Th1 and Th2 cells can be disturbed by a variety of factors, including heavy metals; a shift to a Th2 phenotype has been correlated with the development of allergic responses and some autoimmune diseases (Harber et al. 2000; Mosmann and Sad 1996). As part of our efforts, cytokine gene expression was studied in CD4 cells to investigate the DU-induced Th2 shift hypothesis.

Our array data showed an approximately 2-fold induction of IL-5 expression in CD4^+^ T cells and 1.7-fold induction of IL-10 in macrophages upon DU exposure (Tables 2, 3). We postulate that the reason changes in TNF and IL-6 were not detected, as reported by Krocova et al. (2000), is because of differences in exposure conditions and cell type (we used mouse primary peritoneal macrophages vs. rat alveolar macrophages and lung fibroblasts). However, the conclusions were similar because *IL-10* and *IL-5* were found to be up-regulated, and both genes are of the Th2 type. IL-5 is a signature cytokine of Th2 cells, which also produce cytokines such as IL-4 and IL-10 (Cousins et al. 2002; Mazzarella et al. 2000). IL-10 can also create a microenvironment to facilitate Th2 cell development (Malefyt et al. 1991; Mosmann and Sad 1996). Therefore, up-regulation of *IL-5* and *IL-10* expression in our study indicates a Th2 differentiation tendency after DU exposure. This is direct evidence, at the transcriptional level, for a DU-induced Th2 shift. Th2 domination of T-helper cell population differentiation is often found in association with strong antibody (e.g., autoimmune diseases) and allergic responses (Harber et al. 2000; Mosmann and Sad 1996). Interestingly, elevated blood IL-10 concentrations have been detected in symptomatic Gulf War veterans who were potentially exposed to DU under battlefield conditions (Skowera et al. 2004; Zhang et al. 1999). The data in our study show that DU may contribute to an increase in IL-10 levels through its action on macrophages. Additionally, the induction of IL-5 expression in CD4^+^ T cells, and possibly IL-10 in macrophages suggests an important role for DU in promoting Th2 shifting.

## Conclusions

In summary we have demonstrated DU-induced apoptosis and necrosis in both peritoneal macrophages and splenic CD4^+^ T cells in a cell-specific and concentration-dependent manner. Short-term DU exposure (> 200 μM) to macrophages interferes with the interplay between macrophages and CD4^+^ T cells, resulting in an enhanced T-cell proliferation response. At lower (noncytotoxic) concentrations, DU has the potential to influence immune function by modulating cytokine gene expression mainly involved in signal transductions, interleukin production, chemokine and chemokine receptors, and neurotrophic factors. Array analyses have successfully identified differentially regulated genes implicating DU in carcinogenesis and the development of autoimmune diseases. The up-regulation of *IL-5* and *IL-10* genes in CD4^+^ T cells and macrophages, respectively, strongly suggests a DU-induced Th2 shift during naive T-cell differentiation. Considering the substantial sequence homology between the mouse and human genome and the conserved expression patterns of orthologs reflecting common physiologic functions in these two organisms (Su et al. 2002), the alteration in immune functions and cytokine gene expression in murine immune cells demonstrated in this study identify putative molecular targets for the toxic actions of DU and suggest molecular mechanisms for the development of DU-related diseases in humans.

## Figures and Tables

**Table 1:** Primer sequences used in quantitative RT-PCR for differentially expressed genes

Target	Sequences	Amplicon size (bp)
*IL-10*	F: 5′ -CAT GGG TCT TGG GAA GAG AA-3′	
	R: 5′-CAT TCC CAG AGG AAT TGC AT-3′	194
*Mdk*	F: 5′-ACC GAG GCT TCT TCC TTC TC-3′	
	R: 5′-GGC TCC AAA TTC CTT CTT CC-3′	230
*BMP-11*	F: 5′-TTC ATG GAG CTT CGA GTC CT-3′	
	R: 5′-AGC ATG TTG ATT GGG GAC AT-3′	299
*c-jun*	F: 5-TGA GAA CTT GAC TGG TTG CG-3′	
	R: 5′-AAA GTC CAT CGT TCT GGT CG-3′	222
*Stat-1*	F: 5′-TGG TGA AAT TGC AAG AGC TG-3′	
	R: 5′-TGT GTG CGT ACC CAA GAT GT-3′	119
*Tlr6*	F: 5′-ACA CAA TCG GTT GCA AAA CA-3′	
	R: 5′-GGA AAG TCA GCT TCG TCA GG-3′	128
*IL-5*	F: 5′-GTC CCT ACT CAT AAA AAT CAC CA-3′	
	R: 5′-GAA TAG CAT TTC CAC AGT ACC C-3′	105
*GAPDH*^a^	F: 5′-TGA TGA CAT CAA GAA GGT GGT GAA G-3′	
	R: 5′-TCC T TG GAG GCC ATG TAG GCC AT-3′	240

Abbreviations: F, forward primer; R, reverse primer.

a Sequences are from Lee et al. (2000).

**Table 2:** Differentially regulated genes in DU-exposed peritoneal adherent macrophages as determined by array analysis.

Gene abbreviation^a^	Gene symbol^a^	Accession no.^b^	Gene description	Gene group	*Z*-Test^c^	Ratio^d^	95% CI^e^
Up-regulated gene expression							
*NF-kB p65*	*Rela*	NM_009045	avian reticuloendotheliosis viral (v-rel) oncogene homolog A	Signal transduction	5.8 × 10^–8^	3.9	6.3–2.4
*LTBP-2*	*Ltbp2*	AF004874	latent TGF-beta binding protein-2	Binding protein	1.8 × 10^–6^	3.2	5.3–2.0
*WNT-8b*	*Wnt8b*	NM_011720	wingless related MMTV integration site 8b	Developmental factors	9.3 × 10^–6^	3.2	5.4–1.9
*Mdk*	*Mdk*	NM_010784	midkine	Neurotrophic group	1.5 × 10^–5^	3.1	5.2–1.9
*c-jun*	*Jun*	NM_010591	Jun oncogene	Signal transduction	7.1 × 10^–5^	3.2	5.8–1.8
*Map3k14*	*Nik*—pending	NM_016896	Nfkb inducing kinase	Apoptosis related	1.5 × 10^–3^	2.4	4.0–1.4
*BDNF*	*Bdnf*	NM_007540	brain derived neurotrophic factor	Neurotrophic group	2.4 × 10^–3^	1.7	2.5–1.2
*SOCS-1*	*Cish1*	NM_009896	cytokine inducible SH2-containing protein 1	Signal transduction	2.8 × 10^–3^	2.4	4.2–1.4
*c-myc*	*Myc*	NM_010849	myelocytomatosis oncogene	Signal transduction	7.2 × 10^–3^	2.2	3.8–1.2
*NSG1*/*p21*	*Nsg1*	NM_010942	neuron specific gene family member 1	Signal transduction	7.8 × 10^–3^	2.0	3.3–1.2
*IL-10*	*Il10*	NM_010548	interleukin 10	Interleukin	8.2 × 10^–3^	1.7	2.5–1.2
*Stat 1*	*Stat1*	NM_009283	signal transducer and activator of transcription 1	Signal transduction	9.2 × 10^–3^	2.0	3.4–1.2
*IL-12 Rb1*	*Il12rb1*	NM_008353	interleukin 12 receptor, beta 1	Interleukin receptor	1.1 × 10^–2^	1.6	2.4–1.1
*CIS3*	*Cish3*	NM_007707	cytokine inducible SH2-containing protein 3	Signal transduction	1.1 × 10^–2^	1.8	2.8–1.1
*BMP-9*	*Bmp9*	AF188286	bone morphogenetic protein 9	TGF-beta family	1.4 × 10^–2^	1.8	2.7–1.1
*SMAD7*	*Madh7*	NM_008543	MAD homolog 7 (Drosophila)	Signal transduction	1.9 × 10^–2^	1.6	2.5–1.1
*Tlr2*	*Tlr2*	AF185284	toll-like receptor 2	Cell surface protein	1.9 × 10^–2^	1.8	2.8–1.1
*CT-1*	*Ctf1*	NM_007795	cardiotrophin 1	Cytokine and receptors	1.9 × 10^–2^	1.9	3.4–1.1
*EphA3*	*Epha3*	M68513	mouse eph-related receptor tyrosine kinase (Mek4)	Eph family	2.1 × 10^–2^	1.6	2.3–1.1
*MMP-13*	*Mmp13*	NM_008607	matrix metalloproteinase 13	Protease or related factor	2.2 × 10^–2^	1.9	3.3–1.1
*BMP-11*	*Gdf11*	AF092734	growth/differentiation factor 11	TGF-beta family	3.2 × 10^–2^	1.8	3.1–1.0
*GDNF*	*Gdnf*	D49921	glial cell line-derived neurotrophic factor (GDNF)	Neurotrophic group	3.5 × 10^–2^	1.6	2.4–1.0
*Itgb7*	*Itgb7*	M95632	integrin beta-7 subunit	Intergrin	2.9 × 10^–2^	1.7	2.9–1.0
*MMP-8*	*Mmp8*	NM_008611	matrix metalloproteinase 8	Protease or related factor	4.9 × 10^–2^	1.6	2.5–1.0
Down-regulated gene expression							
*Tlr6*	*Tlr6*	NM_011604	toll-like receptor 6	Cell surface protein	2.7 × 10^–2^	0.6	0.9–0.4
*Cox-2*	*Ptgs2*	NM_011198	prostaglandin-endoperoxide synthase 2	Apoptosis related	2.3 × 10^–2^	0.6	0.9–0.4
*VCAM-1*	*Vcam1*	NM_011693	vascular cell adhesion molecule 1	Adhesion molecule	1.2 × 10^–2^	0.5	0.9–0.3
*FLIPL*	*Cash*	NM_009805	caspase homolog	Apoptosis related	1.1 × 10^–2^	0.6	0.9–0.4
*SARP-1*	*Sdf5*	NM_009144	stromal cell derived factor 5	Apoptosis related	7.3 × 10^–4^	0.6	0.8–0.4

Results are from three individual experiments.

a From Sigma-Genosys (http://www.sigmaaldrich.com/catalog/search/ProductDetail/GENOSYS/G2041).

b From GenBank (http://www.ncbi.nlm.nih.gov/Genbank/).

c *p*-Values in the two-tailed *Z*-tests for the comparison between control and DU treatments.

d The ratio expression values for the average expression values of each gene between DU and control, that is, ratio = intensity value from DU-treated cells divided by that from control cells.

e Confidence intervals (CIs) determined for ratios, *p* < 0.05.

**Table 3:** Differentially regulated genes in DU-exposed CD4^+^ T cells as determined by array analysis.

Gene abbreviation^a^	Gene symbol^a^	Accession no.^b^	Gene description	Gene group	*Z*-Test^c^	Ratio^d^	95% CI^e^
Up-regulated gene expression							
*TECK*/*CCL25*	*Scya25*	NM_009138	small inducible cytokine A25	Chemokine	1.5 × 10^–14^	4.2	6.2–2.9
*Mdk*	*Mdk*	NM_010784	midkine	Neurotrophic group	2.9 × 10^–7^	3.0	4.7–1.9
*IL-5*	*Il5*	NM_010558	interleukin 5	Interleukin	9.5 × 10^–4^	1.9	2.9–1.3
*VEGF-A*	*Vegf*	NM_009505	vascular endothelial growth factor	Angiogenic factor	2.5 × 10^–5^	1.8	2.2–1.4
Down-regulated gene expression							
*EphA3*	*Epha3*	M68513	eph-related receptor tyrosine kinase (Mek4)	Eph family	4.0 × 10^–8^	0.5	0.6–0.4
*CCR-4*	*Cmkbr4*	NM_009916	chemokine (C-C) receptor 4	Chemokine receptor	1.8 × 10^–5^	0.6	0.8–0.5
*LIF*	*Lif*	NM_008501	leukemia inhibitory factor	Cytokine and receptors	3.0 × 10^–5^	0.6	0.8–0.4
*GDF-7*	*Gdf7*	U08339	BALB/c putative growth factor GDF7 (Gdf7) gene	TGF-beta superfamily	2.4 × 10^–4^	0.5	0.7–0.3
*EBF*	*Ebf*	NM_007897	early B-cell factor	Signal transduction	3.7 × 10^–4^	0.5	0.7–0.4
*CD27/TNFRSF7*	*Tnfrsf7*	L24495	CD27 antigen	TNF superfamily	6.4 × 10^–4^	0.6	0.8–0.4
*SLIT-3*	*Slit3*	AF088902	SLIT1 protein	Developmental factor	9.2 × 10^–4^	0.7	0.8–0.5
*CX3CL*1	*Scyd1*	NM_009142	small inducible cytokine subfamily D, 1	Chemokine	1.2 × 10^–3^	0.4	0.7–0.2
*SMAD1*	*Madh1*	NM_008539	MAD homolog 1 (*Drosophila*)	Signal transduction	1.3 × 10^–3^	0.6	0.8–0.5
*A1*	*Bcl2a1a*	L16462	hemopoietic-specific early response protein	Apoptosis related	1.5 × 10^–3^	0.6	0.8–0.4

Results are from three individual experiments.

a From Sigma-Genosys (http://www.sigmaaldrich.com/catalog/search/ProductDetail/GENOSYS/G2041).

b From GenBank (http://www.ncbi.nlm.nih.gov/Genbank/).

c *p*-Values in the two-tailed *Z*-tests for the comparison between control and DU treatments.

d The ratio expression values for the average expression values of each gene between DU and control, that is, ratio = intensity value from DU-treated cells divided by that from control cells.

e 95% Confidence intervals (CIs) determined for ratios, *p* < 0.05.

**Table 4:** Comparison of the gene expression ratios in macrophages determined by microarray and quantitative RT-PCR analysis.

Gene abbreviation	Array ratios	RT-PCR ratio^c^
*Mdk*^a^	3.1	3.2
*c-jun*^a^	3.2	1.9
*BMP-11*^a^	1.8	1.6
*Stat-1*^a^	2.0	2.2
*IL-10*^a^	1.7	6.9
*Tlr6*^a^	0.6	0.6
*Mdk*^b^	3.0	1.9
*IL-5*^b^	1.9	3.2

a The gene was differentially expressed in macrophages.

b The gene was differentially expressed in CD4^+^ T cells.

c Results from triplicate s (*n* = 3).

## References

[b1-ehp0114-000085] Baldwin AS (2001). The transcription factor NF-κ B and human disease. J Clin Invest.

[b2-ehp0114-000085] Benjamini Y, Hochberg Y (1995). Controlling the false discovery rate: a practical and powerful approach to multiple testing. J R Stat Soc Ser.

[b3-ehp0114-000085] Bertho AL, Santiago MA, Coutinho SG (2000). Flow cytometry in the study of cell death. Mem Inst Oswaldo Cruz Rio de Janerio.

[b4-ehp0114-000085] Bierhaus A Nawroth PP2003. From bench to bedside: new roles of NF-κ B. In: Annals of Hematology: 47th Annual Meeting of the GTH (Mannhalter C, Lechner K, Knobl P, Pabinger I, Rintelen C,eds). New York:Springer Press, 103.

[b5-ehp0114-000085] Chen F, Castranova V, Shi X (2001). New insights into the role of nuclear factor-kB in cell growth regulation. Am J Pathol.

[b6-ehp0114-000085] Coligan JE 1991. Proliferative assays for T cell function. In: Current Protocols in Immunology, Vol 1 (Coligan JE, Kruisbeek AM, Margulies DH, Shevach EM, Strober W, eds). Hoboken, NJ:John Wiley & Sons, unit 3.12. 10.1002/0471142735.im0312s60 18432927

[b7-ehp0114-000085] Cousins DJ, Lee TH, Staynov DZ (2002). Cytokine coexpression during human Th1/Th2 cell differentiation: direct evidence for coordinated expression of Th2 cytokines. J Immunol.

[b8-ehp0114-000085] Domingo JL (1995). Chemical toxicity of uranium. Toxicol Ecotoxicol News.

[b9-ehp0114-000085] Domingo JL (2001). Reproductive and developmental toxicity of natural and depleted uranium: a review. Reprod Toxicol.

[b10-ehp0114-000085] Edison AF (1994). The effect of solubility on inhaled uranium compound clearance: a review. Health Phys.

[b11-ehp0114-000085] Fisenne IM, Welford GA (1986). Natural U concentration in soft tissues and bone of New York city residents. Health Phys.

[b12-ehp0114-000085] Furuya R, Kumagai H, Hishida A (1997). Acquired resistance to rechallenge injury with uranyl acetate in LLC-PK-1 cells. J Lab Clin Med.

[b13-ehp0114-000085] Garver RI, Milner PG (1993). Reciprocal expression of pleiotrophin and midkine in normal versus malignant lung tissues. Am J Respir Cell Mol Biol.

[b14-ehp0114-000085] Gazin V, Kerdine S, Grillon G, Nizard P, Bailly I, Pallardy M (2002). Uranium and pulmonary inflammatory response: study of the molecular mechanisms involved in the induction of TNF-α secretion by macrophages. Ann Occup Hyg.

[b15-ehp0114-000085] Gazin V, Kerdine S, Grillon G, Pallardy M, Raoul H (2004). Uranium induces TNF-αsecretion and MAPK activation in a rat alveolar macrophage cell line. Toxicol Appl Pharmacol.

[b16-ehp0114-000085] GEO 2005. Array Data Stored in Gene Expression Omnibus. Bethesda, MD:National Institutes of Health. Available: http://www.ncbi.nlm.nih.gov/geo/[accessed 15 August 2005].

[b17-ehp0114-000085] Harber M Sundstedt A Wraith D 2000. The Role of Cytokines in Immunological Tolerance Potential for Therapy. Cambridge, UK:Cambridge University Press. 10.1017/S1462399400002143 14585135

[b18-ehp0114-000085] Hass JR, Bailey EH, Purvis OW (1998). Bioaccumulation of metals by lichens: uptake of aqueous uranium by *Peltigera membranacea* as a function of time and pH. Am Mineral.

[b19-ehp0114-000085] Hu J, Higuchi I, Yoshida Y, Shiraishi T, Osame M (2002). Expression of midkine in regenerating skeletal muscle fibers and cultured myoblasts of human skeletal muscle. Eur Neurol.

[b20-ehp0114-000085] Kalinich JF, McClain DE (2001). Staining of intracellular deposits of uranium in cultured murine macrophages. Biotech Histochem.

[b21-ehp0114-000085] Kalinich JF, Ramakrishnan N, Villa V, McClain DE (2002). Depleted Uraniumuranyl chloride induces apoptosis in mouse J774 macrophages. Toxicol.

[b22-ehp0114-000085] Krocova Z, Macela A, Kroca M, Hernychova L (2000). The immunomodulatory effect(s) of lead and cadmium on the cells of immune system *in vitro*. Toxicol In Vitro.

[b23-ehp0114-000085] Lee CZ, Royce FH, Denison MS, Pinkerton KE (2000). Effect of *in utero* and postnatal exposure to environmental tobacco smoke on the developmental expression of pulmonary cytochrome P450 monooxygenases. J Biochem Mol Toxicol.

[b24-ehp0114-000085] Leggett RW (1989). The behavior and chemical toxicity of U in the kidney: a reassessment. Health Phys.

[b25-ehp0114-000085] Li M, Carpio DF, Zheng Y, Bruzzo P, Singh V, Ouaaz F (2001). An essential role of NF-κ B /toll-like receptor pathway in induction of inflammatory and tissue-repair gene expression by necrotic cells. J Immunol.

[b26-ehp0114-000085] Lin RH, Wu LJ, Lee CH, Lin-Shiau SY (1993). Cytogenetic toxicity of uranyl nitrate in Chinese hamster ovary cells. Mutat Res.

[b27-ehp0114-000085] Malefyt RDW, Abrams J, Bennett B, Figdor CG, de Vries JE (1991). Interleukin 10 (IL-10) inhibits cytokine synthesis by human monocytes: an autoregulatory role of IL-10 produced by monocytes. J Exp Med.

[b28-ehp0114-000085] Malenchenko AF, Barkun NA, Guseva GF (1978). Effect of uranium on the induction and course of experimental autoimmune orchitis and thyroiditis. J Hyg Epidemiol Microbiol Immunol.

[b29-ehp0114-000085] Mazzarella G, Bianco A, Catena E, De Palma R, Abbate GF (2000). Th1/Th2 lymphocyte polarization in asthma. Allergy.

[b30-ehp0114-000085] McClain DE, Benson KA, Dalton TK, Ejnik J, Emond CA, Hodge SJ (2001). Biological effects of embedded depleted uranium (DU): summary of the Armed Force Radiobiology Research Institute research. Sci Total Environ.

[b31-ehp0114-000085] Miller AC, Blakely WF, Livengood D, Whittaker T, Xu J, Ejnik JW (1998). Transformation of human osteoblast cells to the tumorigenic phenotype by depleted uranium-uranyl chloride. Environ Health Perspect.

[b32-ehp0114-000085] Miller AC, Brooks K, Smith J, Page N (2004). Effect of the militarily-relevant heavy metals, depleted uranium and heavy metal tungsten-alloy on gene expression in human liver carcinoma cells (HepG2). Mol Cell Biochem.

[b33-ehp0114-000085] Mosmann TR, Sad S (1996). The expanding universe of T-cell subsets: Th1, Th2 and more. Immunol Today.

[b34-ehp0114-000085] Moss MA 1985. Chronic low level uranium exposure via drinking water—clinical investigations in Nova Scotia [Master’s Thesis]. Halifax, Nova Scotia:Dalhousie University.

[b35-ehp0114-000085] Muramatsu, T (2002). Midkine and pleiotrophin: two related proteins involved in development, survival, inflammation and tumorigenesis. J Biochem.

[b36-ehp0114-000085] Murray VSG, Bailey MR, Spratt BG (2002). Depleted uranium: a new battlefield hazard. Lancet.

[b37-ehp0114-000085] Pallardy M, Biola A, Lebrec H, Breard J (1999). Assessment of apoptosis in xenobiotic- induced immunotoxicity. Methods (Orlando).

[b38-ehp0114-000085] Pellmar TC, Fuciarelli AF, Ejnik JW, Hamilton M, Hogan J, Strocko S (1999). Distribution of uranium in rats implanted with depleted uranium pellets. Toxicol Sci.

[b39-ehp0114-000085] Pfaffl MW (2001). A new mathematical model for relative quantification in real-time RT- PCR. Nucleic Acids Res.

[b40-ehp0114-000085] Pfaffl MW, Horgan GW, Dempfle L (2002). Relative expression software tool (REST©) for group-wise comparison and statistical analysis of relative expression results in real-time PCR. Nucleic Acids Res.

[b41-ehp0114-000085] Pollard KM, Landberg GP (2001). The in vitro proliferation of murine lymphocytes to mercuric chloride is restricted to mature T cells and is interleukin 1 dependent. Int Immunopharmacol.

[b42-ehp0114-000085] Priest ND (2001). Toxicity of depleted uranium. Lancet.

[b43-ehp0114-000085] Rodenburg RJ, Raats JM, Pruijn GJ, van Venrooij WJ (2000). Cell death: a trigger of autoimmunity?. BioEssays.

[b44-ehp0114-000085] Rook GAW, Zumla A (1997). Gulf war syndrome: is it due to a systemic shift in cytokine balance towards a Th2 profile?. Lancet.

[b45-ehp0114-000085] Shen X, Lee K, Konig R (2001). Effects of heavy metal ions on resting and antigen-activated CD4^+^ T cells. Toxicology.

[b46-ehp0114-000085] Sigma 2004. Panorama Mouse Cytokine Array. St. Louis, MO: Sigma-Genosys. Available: http://www.sigmagenosys.com/epp_mammalian_mc.asp [accessed 15 August 2005].

[b47-ehp0114-000085] Skowera A, Hotopf M, Sawicka E, Varela-CalvinoR, Unwin C, Nikolaou V (2004). Cellular immune activation in Gulf War veterans. J Clin Immunol.

[b48-ehp0114-000085] Su AI, Cooke MP, Ching KA, Hakak Y, Walker JR, Wiltshire T (2002). Large-scale analysis of the human and mouse transcriptomes. Proc Natl Acad Sci USA.

[b49-ehp0114-000085] Tak PP, Firestein GS (2001). NF-κ B: a key role in inflammatory diseases. J Clin Invest.

[b50-ehp0114-000085] Taulan M, Paquet F, Maubert C, Delissen O, Demaille J, Romey M (2004). Renal toxicogenomic response to chronic uranyl nitrate insult in mice. Environ Health Perspect.

[b51-ehp0114-000085] Tully DB, Collins BJ, Overstreet JD, Smith CS, Dinse GE, Mumtaz MM (2000). Effects of arsenic, cadmium, chromium, and lead on gene expression regulated by a battery of 13 different promoters in recombinant HepG2 cells. Toxicol Appl Pharmacol.

[b52-ehp0114-000085] Wooten MW (1999). Function for NF-κ B in neuronal survival: regulation by atypical protein kinase C. J Neurosci Res.

[b53-ehp0114-000085] Wrenn ME, Durbin PW, Howard B, Lipsztein J, Rundo J, Still ET (1985). Metabolism of ingested U and Ra. Health Phys.

[b54-ehp0114-000085] Xu LG, Shu HB (2002). TNFR-associated factor-3 is associated with BAFF-R and negatively regulates BAFF-R-mediated NF-κ B activation and IL-10 production. J Immunol.

[b55-ehp0114-000085] Yamada H, Koizumi S (2002). DNA microarray analysis of human gene expression induced by a non-lethal dose of cadmium. Ind Health.

[b56-ehp0114-000085] Zamora ML (1998). Chronic ingestion of uranium in drinking water: a study of kidney bioeffects in humans. Toxicol Sci.

[b57-ehp0114-000085] Zhang QW, Zhou XD, Denny T, Ottenweller JE, Lange G, LaManca JJ (1999). Change in immune parameters seen in Gulf War veterans but not in civilians with chronic fatigue syndrome. Clin Diagn Lab Immunol.

[b58-ehp0114-000085] Zhou LR, Zhou JH, Yang JC (1998). Effects of cytokines induced by mineral dust on lung fibroblasts *in vitro*. J Occup Health.

